# Large-scale narrative events in popular cinema

**DOI:** 10.1186/s41235-019-0188-x

**Published:** 2019-09-18

**Authors:** James E. Cutting, Kacie L. Armstrong

**Affiliations:** Department of Psychology Uris Hall, Cornell University, 109 Tower Road, Ithaca, NY 14853-7601 USA

**Keywords:** Events, Movies, Narrative, Scenes, Segmentation, Vigilance

## Abstract

Most experiments in event perception and cognition have focused on events that are only a few minutes in length, and the previous research on popular movies is consistent with this temporal scope. Scenes are generally between a few seconds and a few minutes in duration. But popular movies also offer an opportunity to explore larger events—variously called acts, major parts, or large-scale parts by film theorists—in which the boundaries often have few if any unique physical attributes. These units tend to be between about 20 to 35 min in duration. The present study had observers watch seven movies they had not seen before and, over the course of several days and with ample justifications, reflect on them, and then segment them into two to six parts with the aid of a running description of the narrative. Results showed consistency across viewers’ segmentations, consistency with film-theoretic segmentations, and superiority over internet subjects who had access to only the scenarios used by the movie viewers. Thus, these results suggest that there are large scale events in movies; they support a view that their events are organized meronomically, layered with units of different sizes and with boundaries shared across layers; and they suggest that these larger-scale events can be discerned through cognitive, not perceptual, means.

## Significance

Although psychologists admit that events can span essentially any length of time, most research in event perception and cognition has had participants segment events of a few minutes in duration or less. Moreover, current views suggest that events are nested meronomically; that is, smaller events exist within larger events and, more particularly, the larger events share boundaries with the first and last of the smaller events within their scope. The study of popular movie structure allows three queries to test event theory. (1) There is ample psychological evidence for movie scenes as events. For example, in the spirit of Aristotle they can be shown to have beginnings, middles, and ends. These scenes can last from a few seconds to several minutes. Can one find psychological evidence for a movie unit larger than scenes? (2) If so, do they share endpoints with the bordering scenes they subsume? (3) Event theory suggests that smaller units are driven by physical information in the stimuli, and the evidence from movies is consistent with this. But the event theory also suggests that large events are discerned more through cognitive means. Is this true for larger-scale events in movies as well? Results of the segmentation of seven popular movies released between 1927 and 2011 are consistent with the notions that there are larger-scale events in movies between about 20 and 35 min in length, that they share boundaries with the scenes/events they subsume, and that cognitive skills are necessary to segment them.

## Background

Our experience is filled with units of different sizes. We may surf the internet within the task of writing an email letter, within the span of using our laptop, within a bus trip, within a vacation, and beyond to within the span of a particular employment, to within a career. Each of these can be called a unit of experience. Life is not only “one thing after another” (Keillor, 2013, 8:6; Radvansky, 2012, p. 269) but it is also a layered set of sequential “things” one after another, the larger encompassing the smaller.

Many of these things are called *events*, and the criterion for an event is that it must be perceived or thought of as a unit, one that typically—following Aristotle—has a beginning, a middle, and an end (Cutting, 1981; Zacks & Tversky, 2001). Importantly in this conception, the boundary of a large event is one generally shared with a smaller event, and perhaps one still smaller. That is, a smaller event does not typically lie astride the boundary of two superordinate events. Moreover, in contrast to a hierarchy, this structure is called a meronomy; Zacks and Tversky (2001) called it a partonomy. That is, lower-order units are parts of, not types of, higher-order units.

Most temporal art forms are also meronomical, constructed with layers of units of different sizes. A symphony often has four parts (a common structure is sonata, adagio, scherzo, and allegro), each part with a number of sections. The sonata form typically has an exposition, a development, a recapitulation, and an optional coda. And the exposition will often have several main melodic themes that later repeat. Similarly, in reading a novel one will likely encounter chapters, and paragraphs, and sentences. A play will likely have several acts, and scenes within them, and spoken parts and actions within those. A dance, whether choreographed or improvised, will typically have parts within parts. Moreover, the layered structure of movies is roughly the same. Indeed, most of us are accustomed to thinking of movies as having shots within scenes and sequences within the movie. But is there another unit larger than the scene or sequence and smaller than a movie? Indeed, Gibson (1979), p. 391 suggested that a “film is composed of events and superordinate events.” What might these superordinate events be?

Among movie units, shots may seem to be events but they are typically not (Magliano & Zacks, 2011). When asked to detect cuts in a scene, viewers often miss them (Smith & Henderson, 2008) and the later visual areas of the brain take little notice of cuts (Baldassano et al., 2017; Zacks, Speer, Swallow, & Maley, 2010). Thus, although some shots are full scenes, most are not, and they have little status as events in the larger flow of a movie. Scenes are the first units of movies that meet the various criteria for events—they have a normatively scalloped shape in terms of shot duration (longer, then shorter, then longer) and shot scale (wider angle, then perhaps a closeup, then often backing off; Cutting, Brunick, & Candan, 2012) and they have a characteristic brain response, registering the unpredictability of action at a scene boundary but less so within a scene (Zacks, Speer, Swallow, Braver, & Reynolds, 2007). Similarly, sequences are events—made up of smaller scene-like units that have their boundaries disguised and typically oscillate between two characters, two places, or two time frames (Cutting, 2019a).

One candidate for Gibson’s “superordinate event” in movies is analogous to the *act* in a play. Indeed, Field (2005) has called them acts. However, Bordwell (2006) and Thompson (1999) have simply called them large-scale parts, and Bellour (1976) called them major parts, for the obvious reason that the term *act* can have misleading implications. The end of an act in a play may completely halt the action (often with a curtain lowered) whereas no analogous thing occurs in most movies.

However, insofar as we know, no psychological evidence supports a larger act-like unit in movies. To be sure, there are ample theoretical and pragmatic statements about such structures, but without psychological support such theories are, well, just theories. Since events are psychological units they should have psychological evidence in their support. The same should be true of “superordinate events”. The goal of this article is to assess whether there is psychological evidence from young, nonprofessional viewers in support of the larger units. Movies are a good venue for this since, at least in contemporary films, the boundaries between these units are not typically obvious on the basis of their surface form. However, older movies may have fades and dissolves that can assist the viewer in segmentation.

There are two basic approaches to large-scale events in cinema, and both have shared attributes with the works of German novelist and playwright Gustav Freytag (MacEwen, 1900), who proposed a five-part structure for plays, and more importantly with the mythologist Joseph Campbell (1949) and his “hero’s journey”. Campbell proposed a three-part structure having a departure, an initiation, and a return. One approach to the narrative structure of movies comes from screenwriting manuals. Perhaps allied with Campbell, it is most associated with Field (2005) and is known as the three-act structure.

The first quarter of a movie is aptly named the *setup*. In it the main characters are typically introduced and we learn about their goals. There is likely a *turning point* (also called an *inciting incident*) typically about halfway through it, which raises a dramatic question (e.g., will the protagonist outwit the antagonist?) that will be answered at the end of the movie. The second act, the *confrontation*, typically contains the middle half of the movie, in which the protagonist attempts to solve the problem created by the inciting incident. The confrontation has a midpoint, where the protagonist may enlist the help of other characters. The third act is the *resolution* or *climax* where the story and its subplots are resolved and the dramatic question is answered.

The second approach comes from film theory and is not much different. For Bordwell (2006, 2007) and Thompson (1999, 2008) the *setup* and the *climax* are pretty much as Field describes, although the climax may have an epilog in which the diegetic social order is restored. One way in which this approach differs from Field’s is that it divides his second act in half to create two other large-scale parts of roughly the same length as the setup and climax. Its first half (and second large part of the movie) is the *complicating action* in which the protagonist’s goals are sharpened but still not met, and the second half (and third large part) is the *development*, which can create new goals for the protagonist, deepen characterization, enlist minor characters, or simply sustain the situation.

Two important features accompany this approach. First, the parts are generally, but not necessarily, the same length; and second, the number of parts is not limited to four. Instead, there is a rough time limit of 20 to 30 min (Thompson, 1999) or 25 to 35 min (Bordwell, 2007; Thompson, 2008) that will tend to dictate the number of larger parts. Thus, a movie of 90 min or less is likely to have only three parts and no development section, whereas a movie of near 150 min or more is likely to have five parts and two development sections. Thompson (1999), pp. 43–44 believed that balanced-length parts:

cater to the attention span of the spectator. The studios need not have pinpointed exact timings consciously, but careful attention to the minute-by-minute reactions of preview audiences (used since the 1920s) may have given practitioners an instinctive sense of when to change the direction of action. Time and again scriptwriters have described this instinctive feel for structure … These generalizations about the large-scale parts of narratives [however] do not offer a detailed or definitive explanation as to why they exist. Such an explanation could lie in the realm of cognitive psychology.

The purpose of this paper is not to investigate the reason for these 20–35 min parts. Instead, it seems prudent first to determine whether or not these large-scale units in the Thompson/Bordwell scheme are actually psychological events for nonprofessional viewers.

## Experiment 1: Segmentations by average viewers

### Method

As part of a seminar course undergraduate students viewed seven movies as an ensemble, each movie in a single sitting. Rather than being a convenience sample, this is a sample target audience for most popular movies. None of the students had taken a film course, most were computer science or psychology majors, and most reported normally seeing about one movie per week. Thirteen to seventeen viewed each movie around a seminar table in a darkened classroom. Movies were viewed in chronological order by release year, and on average about 10 days apart. There were ten females and seven males in the class.

We chose these movies because they were unlikely to have been seen before (which proved true, no student had seen any of them) and because they had widely varying narrative structures and narrational techniques—single or multiple narrative threads; linear and nonlinear plot lines; considerable differences in shot durations, shot scales, luminances, and motion; their uses (or not) of dissolves and fades; and the presence (or not) of flashbacks. This research was exempted from full review by the Cornell Institutional Review Board and informed consent was obtained.

### The movies

Several attributes of the movies, and of the results, are given in Table 1. Here, we describe each movie in more detail, with information about its general reception and historical context.

**Table 1:** Films and segmentation information

	Film duration	Number of viewers	Number of narrative entries for each scenario	Number of segments given by experts	Mean number of segments per viewer (standard deviation)	Number of film locations endorsed as boundaries by at least three viewers	Mean leave-one-out correlation of viewer congruence (r)	Correlation of viewer aggregate with film theory (r)
*Wings* (1927)	144 min	13	82	4	5.2 (0.8)	8	0.58	0.82
*Grand Hotel* (1932)	112 min	16	42	4	4.4 (0.7)	8	0.37	0.60
*Passage to Marseille* (1944)	109 min	16	61	(4)	4.7 (0.8)	5	0.82	0.89
*Rope* (1948)	80 min	16	68	(3)	4.6 (1.1)	8	0.47	0.67
*All About Eve* (1950)	138 min	17	59	5	4.7 (1.3)	9	0.30	0.66
*Ordinary People* (1980)	124 min	17	73	(4)	4.8 (0.8)	8	0.36	0.56
*Source Code* (2011)	93 min	16	108	3	4.8 (0.5)	10	0.25	0.42

*Wings* (Wellman et al., 1927**)** is a drama/romance/war movie in black and white. It is also a “silent” movie, the only one in this sample. That is, it is accompanied by music but has no voice track. It won Academy Awards for Best Picture and for its technical accomplishments in the portrayals of air battles. Its Internet Movie Database (IMDb) rating is 7.7 and its Rotten Tomatoes ratings are 95% by critics and 78% by general audiences.^1^ It has 2061 shots (1797 live action shots with 264 intertitles), and average shot durations of 3.9 s without intertitles and 5.0 s with them. Silent movies typically have two types of intertitles, conversational and expositional. Expositional intertitles act typically like extended scene transitions and are inserted by the filmmakers to form boundaries among scenes of other narrative units. Among the non-cut transitions between shots in *Wings* are one intermission, 36 dissolves, 37 fades out and in (sometimes in pairs with title cards in between), and two wipes. It also has a linear narrative style with temporal gaps as it progresses. There is only one brief flashback.

*Grand Hotel* (Goulding et al., 1932) is a black-and-white drama, and an early “talkie” (with indigenous sound and dialog). It is perhaps the earliest example of a network narrative in popular cinema (Bordwell, 2015). That is, each of the five major characters has his or her own narrative thread, and these threads crosscut throughout the movie, usually paired with at least one other character. *Grand Hotel* also won the Academy Award for Best Picture; its IMDb rating is 7.5 and its Rotten Tomatoes ratings are 86% from critics and 77% from general audiences. It has only 380 shots, yielding a very long average shot duration of 17.6 s. Among its non-cut transitions are five dissolves, seven fades out and in, and three wipes. It has no flashbacks.

*Passage to Marseille* (Curtiz et al., 1944) is an adventure/drama/war movie in black-and-white. It is a follow-up to *Casablanca* (Curtiz et al., 1942) with much the same cast and again focused on the French resistance in World War II. It has a complex plot. Most notably, it has a flashback within a flashback within a flashback within the dominant diegetic story. Its IMDb rating is 6.9 and has no Rotten Tomatoes critics’ rating (it received almost no reviews at the time in part because it was released when it was already apparent that the Allies would win the war), and an audience rating of only 56%. It has 1039 shots, with average shot duration of 6.2 s. Among its shot transitions are 78 dissolves, five fades out and in, and eight wipes.

*Rope* (Hitchcock et al., 1948) is an experimental drama/suspense movie. Hitchcock regarded it as a stunt and a failure (Truffaut & Scott, 1983, pp. 179–184). It was also his first color movie. It has an IMDb rating of 8.0 and Rotten Tomatoes ratings of 97% from critics and 90% from general audiences. It has only 11 shots (average shot duration = 434 s) and was designed to appear to have (almost) no edits. Edits were included for pragmatic reasons of film-reel length in shooting, and during theatrical presentations to cue the projectionist when to start the next reel. It has five straight cuts and five dissolves (typically across the backs of male characters with dark jackets) and no standard fades or wipes. The story takes place in real time in one room.

*All About Eve* (Mankiewicz et al., 1950) is a black-and-white drama. It won the Academy Award for Best Picture and several other awards. It has an IMDb rating of 8.3, Rotten Tomatoes ratings of 100% from critics and 94% from general viewers, and the American Film Institute has it currently ranked as the 16th best movie of all time. It has 784 shots with average shot duration of 12.9 s. Among its transitions are 16 dissolves, four fades out and in, and no wipes. The bulk of the movie (87%) is embedded in one flashback.

*Ordinary People* (Redford et al., 1980) is a color drama, and also won the Academy Award for Best Picture. Its IMDb rating is 7.8, with Rotten Tomatoes ratings of 90% from critics and 88% from general audiences. It has 1182 shots and average shot duration of 6.1 s. Among its transitions it has nine dissolves in its opening montage, but otherwise no fades, wipes, or other dissolves. It has 42 short flashbacks (average duration of 3.34 s, and a median duration of 2.17 s). Moreover, much of the movie is edited in a parataxic style; that is, there is often little apparent relation or lead in from one scene to the next. Rather, the simple juxtaposition of scenes forces the viewer to figure out some of the narrative threads and connections as the movie progresses.

*Source Code* (Jones et al., 2011) is a color action/science fiction/puzzle film. Puzzle films (see Buckland, 2009) are designed to be complex and break the boundaries of classical plot structure. *Source Code* has an IMDb rating of 7.5 and Rotten Tomatoes ratings of 92% from critics and 82% from audiences. It has 1478 shots, yielding average shot duration of 4.36 s. It has many complex, compound transitions (swirls), but no standard dissolves, fades, or wipes. It also has 27 changes of venue—cycling back and forth between two locales.

### Procedure

Each movie was projected in a classroom with institutional LCD and sound equipment from a laptop computer with mp4 files downloaded from commercial DVDs. The movies differed in aspect ratios (image width/height)—the first five at 1.37 (Academy ratio), the sixth at 1.85 (widescreen), and the last at 2.35 (Cinemascope)—but the width of the projected images on the screen was constant. Its lateral subtense varied according to the position of the viewer in the room from about 45° (comparable to the view from the middle of a standard movie theater) to about 20° (comparable to viewing a movie on a laptop).

Numbered, sequential content summaries (henceforth called scenarios) for each movie were purpose written for these studies. Each entry was concise with a mean description length across movies of seven to twelve words. The number of itemized entries for each movie is given in Table 1. Scenarios were handed out immediately prior to viewing.^2^ As the room was quite dark, only a few looked at these at the time. Across movies, an average of 55% of the entries in these summaries indicated a scene boundary (here, a change in location and/or time; but see Cutting et al., 2012; Polking, 1990); the rest elaborated content continuing within a scene. These data are shown in Table 2 and are discussed later.

**Table 2:** Viewer segmentations at scene and non-scene boundaries

	Total number of segmentations at scene boundaries	Number of scene boundaries in the scenario	Total number of segmentations at non-scene boundaries	Number of non-scene boundaries in the scenario	Two-sample *t* tests of scene and non-scene boundaries	
*Wings* (1927)	51	38	4	44	*t(*80) = 3.27	*p* < 0.0016
*Grand Hotel* (1932)	48	26	5	16	*t*(40) = 2.73	*p* = 0.0094
*Passage to Marseille* (1944)	55	35	4	26	*t*(59) = 1.92	*p* = 0.06
*All About Eve* (1950)	55	31	8	28	*t*(57) = 4.09	*p* < 0.0001
*Ordinary People* (1980)	62	58	0	14	*t*(70) = 2.43	*p* = 0.018
*Source Code* (2011)	51	46	10	64	*t*(108) = 4.72	*p* < 0.0001
SUM	322	234	31	192		

No classroom discussion occurred after the screenings. Given constraints of course scheduling there was no time to do so. Instead, students were encouraged to think independently about the movie over the next days and then, with the aid of the scenario, segment the movie into two to six parts according to their own interpretations of the narrative by placing lines between the particular entry numbers that marked the transition between one narrative part and the next. This range appeared not to constrain results. Across the seven movies the percentages for values of two to six segments were 2, 9, 24, 50, and 15%, respectively. Table 1 gives more specifics for each movie.

Viewers then wrote a three- to five-page essay justifying their divisions. They were explicitly told that there was no correct answer, only justifiable ones. At the time of their viewings, they had not been instructed about various theories of large narrative parts in movies (Field, 2005; Thompson, 1999), although classroom discussion revealed that several were aware of the general notion of a three-act structure (Field, 2005). Given the number of large segments reported for each movie in Table 1, there is clearly more going on in viewers’ responses than simply trying to impose three acts. Essays and segmentations were gathered at the next class; only the segmentations are discussed here. Although we were unable to control possible collaborations, for six of the seven movies none of the segmentation data across all possible pairs of viewers correlated perfectly (0 of a total of 800 comparisons). We discuss the seventh film below.

Each viewer’s segmentations were recorded for each movie on a spreadsheet. Boundaries were entered as corresponding to the numbered entry on the scenario that began a new segment. These values were averaged across entries for all viewers, becoming *y* axis values. Finally, for graphical purposes, the sequential increments along this vector were adjusted to reflect the movie duration corresponding to each entry; thus, becoming *x* axis values. The result was then plotted as boundary agreement (the proportion of viewers indicating each entry as the onset of a new narrative segment) by running time through the movie.^3^ For four of the seven movies these vectors were compared to professional reports: two by Thompson (1999) and two by Bordwell, one from his blog (Bordwell, 2011) and one from personal correspondence. For the other three, we segmented the movies according to the published guidelines and descriptions of Bordwell (2007) and Thompson (1999). We will call all of these *film-theoretic segmentations.*

We will consider the movie narratives and viewers’ corresponding results in the chronological order of the movies’ release dates. In what follows, the duration of each movie (given in Table 1) was normalized to 1.0. Detailed synopses are given in the Appendix. There, segmentation boundaries selected by at least two observers are indicated as a function of the proportional runtime through the movie, and expressed as a proportion of the number of viewers of the movie.

## Results and preliminary discussion

### *Wings*

**Fig. 1:**
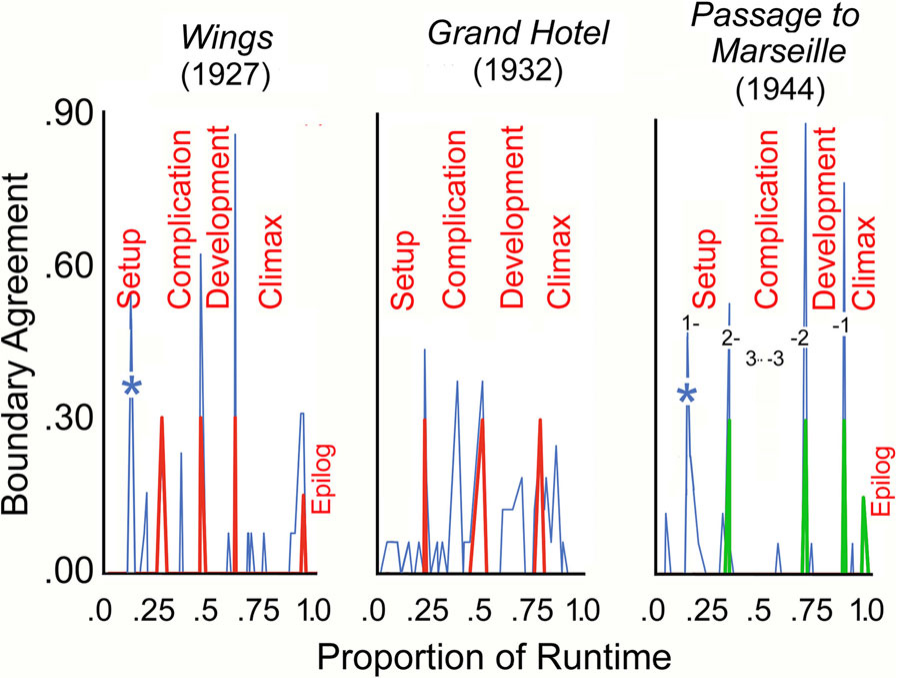
The correspondence between the aggregated viewer responses (proportional agreement that a narrative boundary has occurred) and theoretical considerations in segmenting three early films. The *thin blue spikes* are the viewers’ data; the *thicker red spikes* in the *left* and *middle panels* derive from Thompson (1999), and the *thicker green spikes* derive from descriptions by her and by Bordwell (2006). The width of the base of the spikes is due to two factors. The left flank represents the duration of the last scene before the transition, and the right flank the duration of the scene following it. The *right panel* also has a series of numbers that represent the beginnings and ends of the nested flashbacks

The results for *Wings* are shown in the left panel of Fig. 1. Again, on the ordinate is the segmentation mean (proportion of agreement among viewers that a boundary has occurred), and on the abscissa is the proportion of runtime through the movie. The thin blue lines are the combined viewers’ data and the red, thicker-lined spikes are segmentation points derived from Thompson’s (1999), p. 357 analysis of this movie. We determined these by matching her published segment durations with our time stamps.

**Fig. 2:**
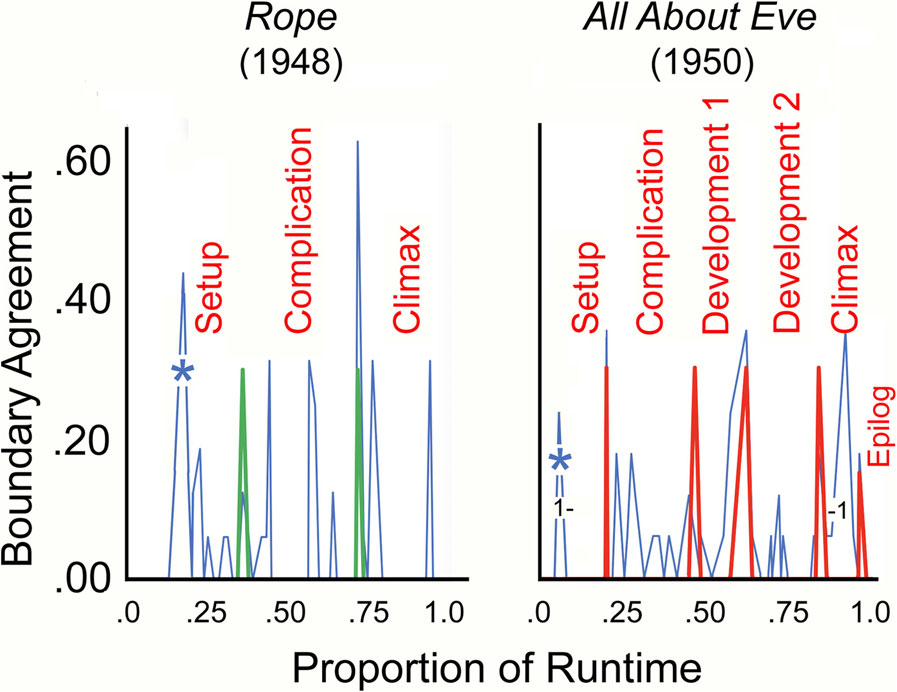
The correspondence between viewer segmentations (agreements that a narrative boundary had occurred; *thin blue spikes*) and those that that follow the rubrics of Thompson (1999) and Bordwell (2006) as *thicker green spikes*, and those provided by Bordwell (personal communication) as *thicker red spikes*. The numbers in the *right panel* denote the time period of the flashback

**Fig. 3:**
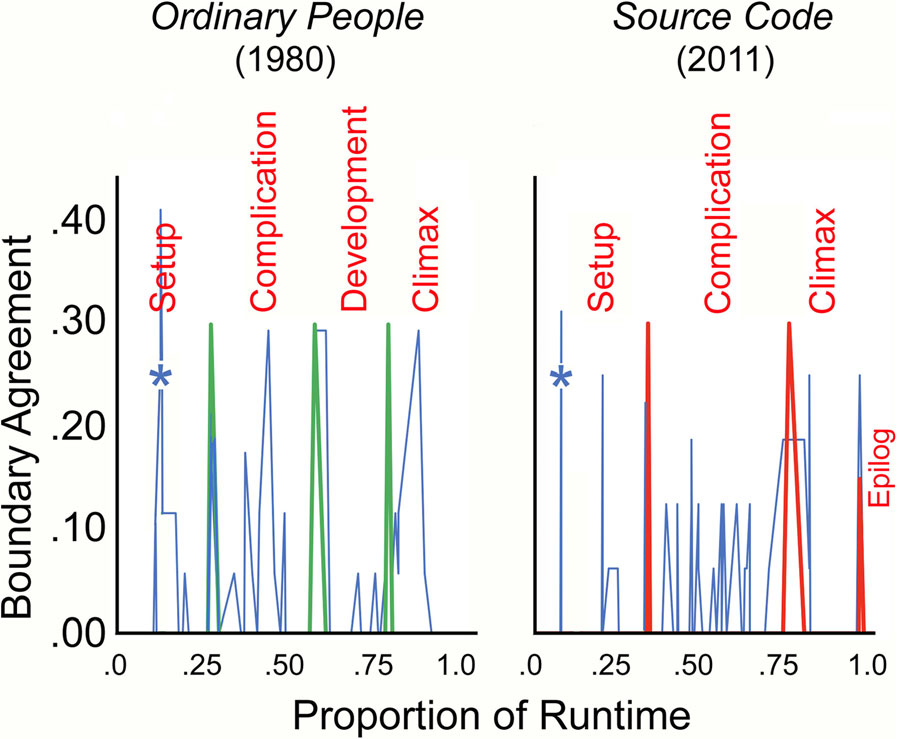
The correspondence between viewer segmentations (*thin blue spikes*) and segmentations for two more recent films that follow the rubrics of Thompson (1999) and Bordwell (2006) in the *left panel*, and that correspond to Bordwell’s (2011) segmentations in the *right panel*

The three main divisions are marked—separating the setup (33 min in length) from the complicating action (29 min; here and elsewhere simply called the *complication*) from the development (27 min)^4^ from the climax (37 min). These breaks are given values akin to 30% agreement so that they can be more easily compared across movies and panels in Figs. 1, 2, and 3. Thompson also designated an epilog for *Wings*. Since she proposed that epilogs are part of the climax, the red spike for the epilog is given a smaller value, here at 15%. Values of zero are given for all other entries. Segmentation points within the narrative and with their particular agreement across viewers are noted in the synopsis in the Appendix.

The mean correlation among viewers’ response patterns was substantial, and is given in Table 1.^5^ Analogous to leave-one-out cross-validation (e.g., Shao, 1993), we correlated each viewer’s segmentation pattern with the average segmentation pattern of all the other viewers across the 82 scenario entries. We then took the mean of those leave-one-out correlations (mean *r* = 0.58, *t* (80) = 6.3, *p* < 0.0001, *d* = 1.4). Moreover, correlating the aggregated viewer data with the Thompson predictions of four large narrative parts plus an epilog shows that the agreement is also quite impressive (*r* = 0.75, *t* (80) = 10.1 *p* < 0.0001, *d* = 2.26). We take this result to be an endorsement of the Thompson/Bordwell theory that traditional popular movies have four large narrative parts with the last (the climax) having an optional epilog.

Yet there are many fades in the movie bracketing an intertitle and these might help viewers segment the narrative. Indeed, we created a 2×2 table for each viewer, with fade transitions that were chosen as boundaries (hits) and non-fade transitions chosen as boundaries (false alarms), versus fades not chosen (misses) and non-fades that were not chosen (correct rejections). We then calculated signal-detection indices corresponding to viewers’ boundaries—mean *d’* = 0.90 (*t* (11) = 5.18, *p* = 0.0003). Thus, and unsurprisingly, the particular transitional information used by the filmmakers could have been used by viewers while encoding the narrative. However, no indication of the transition type (fade, dissolve, cut, or even intertitle content) was given on the scenarios. Thus, by the time the viewers set out to segment the movie, this particular information would almost certainly not be remembered.

### *Grand Hotel*

The results for *Grand Hotel* are shown in the middle panel of Fig. 1. Thompson’s (1999), p. 357 large parts are 24, 32, 27, and 27 min in duration. The viewers’ data show that three of the five largest data peaks occur at locations given by Thompson for this movie, but the results appear a bit less impressive than those for *Wings*. Again, all the peaks in the figure are noted in the synopsis in the Appendix.

The mean leave-one-out correlation of response patterns in the 42 scenario entries across viewers was more modest than that for *Wings*, but still reasonable (mean *r* = 0.37, *t* (40) = 2.42, *p* = 0.02, *d* = 0.77). Overall and again, the pattern of correspondence between the aggregated data and the predictions by Thompson is quite strong (*r* = 0.67, *t* (40) = 5.7, *t* (40) = 5.7, *p* < 0.0001, *d* = 1.8), with three substantial peaks in the data at the boundaries that Thompson assigned.

However and again, there are many fades in the movie and this low-level visual information could have helped some viewers in their segmentations. Signal detection analysis (fades as segment boundaries and not, against non-fades as boundaries and not) again yielded results roughly consistent with their use (mean viewer *d*’ = 0.42, *t* (15) = 2.09, *p* = 0.054). These, of course, were designed by the filmmakers to do exactly that. But again, there was no indication of transition type on the scenario, so this information if relevant could only have been used in storing mental models of the narrative at the time of watching the movie (e.g., Swallow, Zacks, & Abrams, 2009).

### *Passage to Marseille*

The right panel of Fig. 1 shows the data, the large parts, and the numbered flashback locations for *Passage to Marseille.* Shown as green spikes and prior to gathering the data, we segmented the major parts of the movie at points 0.33, 0.70, and 0.88, with a turning point at 0.12 and an epilog at 0.98, yielding large narrative parts of 36, 38, 20, and 13 min. Overall segmentation data are again given with the synopsis in the Appendix.

The leave-one-out correlations among viewer segmentations were very substantial (mean *r* = 0.82, *t* (59) = 14.7, *p* < 0.0001, *d* = 3.8), as were aggregate viewer results to the film-theoretic divisions (*r* = 0.85, *t* (59) = 12.2, *p* < 0.0001, *d* = 3.2). However, it should be no surprise that these results are driven by the many flashback patterns of the movie, noted numerically in the right panel of Fig. 1. Indeed, likely because of these and unlike any of the other movie data in this sample, some of the pairwise segmentation comparisons across viewers yielded perfect correlations (18 of 105).^6^

Clearly, the filmmakers used flashbacks (and their accompanying fades and dissolves) to aid segmentation. Using the 2×2 table of boundaries with and without fades by non-boundaries with and without fades, this information could have been influential in segmentation for individual viewers at the time of encoding (mean *d*’ = 0.53; (*t* (15) = 4.16, *p* = 0.0008). Perhaps because flashbacks dominate viewer segmentations, filmmakers after the 1940s and 1950s decided that the block construction (Bordwell, 2017) of the narrative generated by long flashbacks constrain storytelling too much and stopped using them as often.

### *Rope*

Because it is a short feature film (80 min), *Rope* is a candidate for having only three major sections (Thompson, 1999). The left panel of Fig. 2 shows the response data. Our prior-to-viewing divisions, based on Bordwell’s (2006) and Thompson’s (1999) general descriptions yielded segments of 27, 29, and 23 min in duration. Again, mean segmentations are discussed in the Appendix.

There was considerable correspondence among viewer responses (mean *r* = 0.47, *t* (66) = 4.3, *p* < 0.0001, *d* = 1.06) and between their pooled responses and film-theoretic segmentations (*r* = 0.47, *t* (66) = 4.3, *p* < 0.0001, *d* = 1.06). The importance of both results in this context is that *Rope* has no cuts, fades, or flashbacks that helped with segmentation, and only one dissolve associated with a response peak (which was not a major boundary). Thus, these results must be solely driven by viewers’ cognitive inferences while encoding the story.

### *All About Eve*

*All About Eve* is a long and complex movie as outlined in the Appendix. Fortunately, we were able to enlist David Bordwell, who provided us with an authoritative set of divisions.^7^ He suggested that it was appropriate to divide it into five parts with durations 27, 36, 21, 31, and 21 min. The addition is a second development stage. The correspondences between the viewers’ and his segmentations are shown in the right panel of Fig. 2.

There was adequate correspondence among viewer segmentation patterns (mean *r* = 0.30, *t* (57) = 2.3, *p* = 0.023, *d* = 0.61). And, despite some discrepancies and the dominance of the flashback, the relation between the viewers’ segmentations and Bordwell’s was solid (*r* = 0.58, *t* (57) = 5.38, *p* < 0.0001, *d* = 1.42). However and again, the patterns of fades and dissolves may have played a part in viewers’ responses (mean *d’* = 0.46, *t* (14) = 3.97, *p* = 0.0014).

### *Ordinary People*

The left panel of Fig. 3 shows the data and our segmentations for *Ordinary People*, which created large segments of 32, 36, 26, and 25 min. The correspondence among viewer responses patterns is substantial (mean *r* = 0.36, *t* (71) = 3.2, *p* = 0.002, *d* = 0.76) as is that between viewers’ collective data and our segmentations (*r* = 0.46, *t* (71) = 4.4, *p* < .001, *d* = 1.04). Moreover, as with *Rope*, there were no fades or dissolves to help viewers along the way while watching the movie.

### *Source Code*

Like *Rope*, *Source Code* is a relatively short feature. Thus, Bordwell (2011) segmented it into three parts (33, 34, and 18 min) with a short epilog, as shown with red spikes in the right panel of Fig. 3. As expected for a puzzle film and as indicated in the Appendix, *Source Code* has a complex, nonstandard story. Because there are at least 27 changes back and forth between two diegetic locations—a Chicago commuter train and a Nevada Army laboratory—12 segments of which are more than a few minutes long, many other segmentations were possible. Nonetheless, there is satisfactory correspondence among viewers (mean *r* = 0.25, *t* (106) = 2.63, *p* = 0.01, *d* = 0.51), and a solid correspondence between the mean responses by the viewers and by Bordwell (*r* = 0.44, *t* (106) = 5.04, *p* < 0.0001, *d* = 0.98). Finally, the presence of swirling distortions as transitions between the two venues (train and lab) provided no aid in viewers’ segmentations (mean *d’* = − 0.05).

### Aggregated results

#### Turning points

The correspondence of the viewers’ data with the larger narrative segments proposed by Thompson and Bordwell is quite robust across all seven movies. However, in six of the movies there is reasonable evidence that the viewers also thought that a turning point within the setup also marked an important boundary. Indeed, it was the most prominent boundary in *Ordinary People* and *Source Code*, the second most prominent in *Rope*, and quite substantial in *Wings*, *Passage to Marseille*, and *All About Eve.*

Field (2005) placed emphasis on the concept of an inciting incident (also called a turning point) in the setup; Bordwell (2016) noted that screenwriting manuals typically promote an early inciting incident; and Thompson (2008) has suggested that an inciting incident is one of many turning points. Be that as it may, viewers in this context were clearly influenced by an early turn in the narrative independent of the larger parts that followed. From a screenwriting and filmmaking perspective this is a well-known design feature. Indeed, Ebert (1985) suggested that an inciting incident early in the movie is necessary because of what he called Brotman’s law (named after a Chicago movie exhibitor): “If nothing has happened after the first reel [10 to 12 minutes], nothing is going to happen.” In other words, film practice necessitates some early event that hooks the viewer into the narrative. Waiting until the setup/complication boundary may be too late.

#### Accumulated movie segmentations

**Fig. 4:**
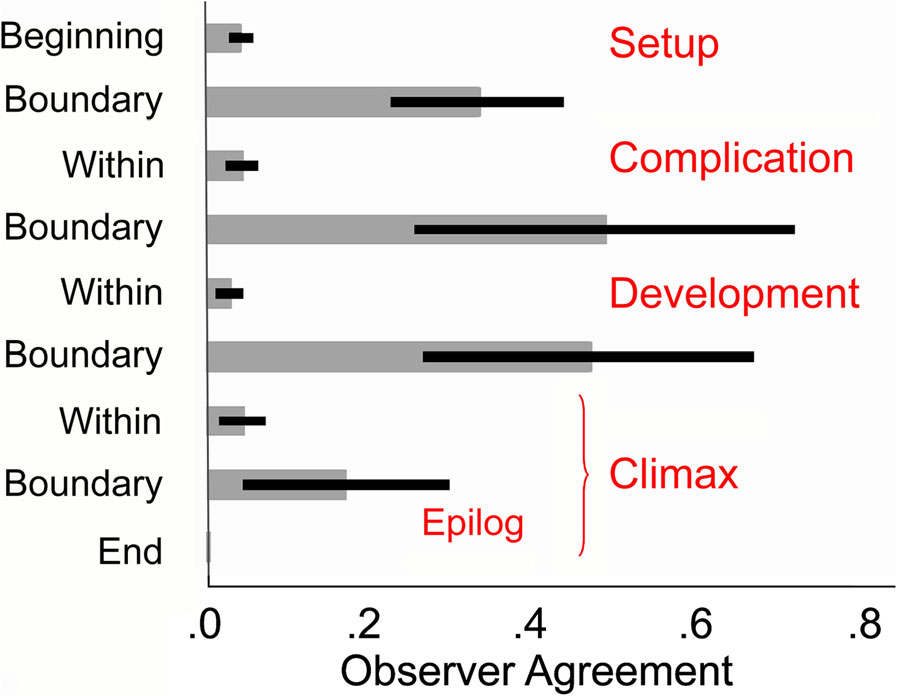
*Gray bars* show the mean interobserver correlations in segmentation performance at nine sections pooled across the seven movies: (1) at all scenario entry boundaries before the end of the setup, (2) at the setup/complication scenario boundary, (3) at all scenario boundaries within the complication, (4) at the complication/development boundary, (5) at all boundaries within the development, (6) at the development/climax boundary, (7) at all boundaries within the climax before the epilog, (8) at the epilog boundary, and (9) at all scenario boundaries after the beginning of the epilog. *Black ribbons* indicate the 95% confidence intervals

We next combined the results from all seven movies by placing viewers’ responses into nine categories: (1) averaging all segment boundaries placed in all entries before the setup, (2) those at the setup/complication boundary, (3) those between that boundary and the complication/development boundary, (4) those at the complication/development boundary, (5) those between that boundary and the development/climax boundary, (6) those at the development/climax boundary, (7) those between that boundary at the beginning of the epilog, if any, (8) those at the beginning of the epilog, if any, (9) and those after the beginning of the epilog. Mean observer agreement and 95% confidence intervals are shown in Fig. 4. The omnibus effect across these nine categories (with films entered as a nominal variable) was robust (*F* (8,43) = 13.4, *p* < 0.0001, η^2^ = 0.67), and contrasting the boundary and within-large-segment results was equally so (*t* (56) = 8.72, *p* < 0.0001, *d* = 2.33). Interestingly, there was no substantial difference among the movies (*F* (6,43) = 1.4, *p* = 0.23).

In addition, since this research is a within-subjects design, we looked at some overall results in the correlations between the viewers’ segmentations on the one hand and the film-theoretic segmentations on the other across the seven movies. Perhaps somewhat surprisingly, there was little evidence for individual differences (*F* (17,87) = 1.32, *p* = 0.20). Our surprise is based on the widespread notion that there is considerable differential appreciation for all forms of entertainment. Indeed, movies in particular are often touted as one of the arenas where people vary greatly (e.g., Chamorro-Premuzic, Kallias, & Hsu, 2013; Rentfrow, Goldberg, & Zilca, 2011). However, one should remember that the ability to segment a movie is not the same thing as liking a movie. Segmentation is a strong correlate of understanding (Sargent et al., 2013), not of affinity.

#### Boundary sharing across event sizes

In addition, and in keeping with a meronomical approach to event cognition, we compared viewers’ responses at points within each film that were scene boundaries (changes in location and/or time) on the scenarios with those that were not. We accumulated boundary judgments across observers at each scene break and non-scene break and compared the two distributions. As shown in Table 2, for the six movies that had scene boundaries (*Rope* does not) there were more segmentations in each movie at scene boundaries than within scenes, and together the aggregate revealed a strong effect (*t* (418) = 7.12, *p* < 0.0001, *d* = 0.70). This result is consistent with the idea that larger events share boundaries with the smaller events that they subsume, which is the criterion for meronomy. An example of a scenario fragment is given in Table 3. It has six non-scene breaks and eleven scene breaks, two of which is a major break in the narrative. Also shown are the location changes, time changes, and the number of segmentations offered by the viewers.

**Table 3:** A fragment of the scenario for *All About Eve* (1950)

Scenario description	Scene break	Major break	Location change	Time change	Total segmentations
Margo [the theater star], upstairs, is dressed for Bill’s party [Bill is her partner and the play’s director]. Bertie [Margo’s housekeeper] helps her tidy her dress	x		x	x	3
Downstairs Bill, Eve [the rising star], and then Margo talk; Eve leaves, Bill & Margo quarrel	x		x		2
Karen [Margo’s best friend], Lloyd [the playwright], & Max [the producer] arrive; odd discussions accrue; Margo says “it’s going to be a bumpy night”					0
Margo is sitting next to piano player. She’s drunk	x			x	1
Max has heartburn; Max & Margo go to kitchen & talk. She offers relief to Max	x		x		1
Lloyd enters kitchen; Max leaves; Lloyd & Margo talk					0
Karen & Eve are upstairs, they talk; Eve wants to be the understudy for Margo and asks her to put in a good word with Lloyd	x		x		1
On the steps: Addison [the theater critic], Bill, Eve, and Karen discuss “the theater”	x		x		0
Margo quarrels with all and goes upstairs; Bill follows later; the rest leave					2
The next day Margo arrives very late to the theater for the understudy audition of Lloyd’s play, in which Margo stars	x	x	x	x	1
Margo learns from Addison that Eve is her understudy					0
Margo enters theater, learns that auditions are over; quarrels with Lloyd, then with Max	x		x		1
Afterwards, Bill and Margo quarrel. Bill says he’s leaving her					0
Later at their home Karen paints, Lloyd comes home furious with Margo; Karen schemes to help Eve	x		x	x	1
That weekend Karen and Margo are stuck in the car without gas (an event that Karen arranged), as Lloyd goes to get some; Margo misses her performance, Eve performs	x		x	x	4
Eve does well; Addison goes to meet her in dressing room; he overhears Bill congratulate her, and she then flirts with Bill; but Bill leaves unimpressed	x	x	x	x	6
Addison and Eve talk; Addison asks questions about her background; Eve is inconsistent and thinks the Shubert Theater is in San Francisco (not New Haven)					1

Note: Words in brackets did not appear in the scenarios of viewers, but only in those of non-viewers

One might worry about the data of Experiment 1. That is, one might suspect that individuals *not having seen a movie* could appropriately segment its narrative simply on the basis of studying the written scenarios. After all, there the narrative is laid out in plain view and with some effort its structure ought to be reasonably discernible. We certainly believed this to be true, but we thought it possible that the responses of non-viewers might not conform to the film-theoretic segmentations as well as those of viewers who had recently seen the movie. Thus, it seemed appropriate to assess the possible differences between segmentations of viewers and non-viewers. Non-viewers were recruited online from Mechanical Turk.

## Methods

Four of the seven movies were selected—*Wings*, *Rope*, *All About Eve*, and *Ordinary People.* In a Qualtrics survey, instructions from Experiment 1 were repeated as closely as possible. To encourage thorough reading the scenarios were modified so that four numbered entries appeared on the display screen at a time, and the workers had to page through the listed entries. Following the last page of entries, they were provided with the same numbered list, but this time the entire list of entries was on one page, and each entry had an empty check box to the left of it. Workers were instructed to segment the film into two to six parts by checking the boxes next to the entries that they believed began a new segment of the film. To ensure that they thoughtfully completed the task, they were also instructed to provide a brief narrative summary of each segment in text boxes provided below the complete list of entries.

**Fig. 5:**
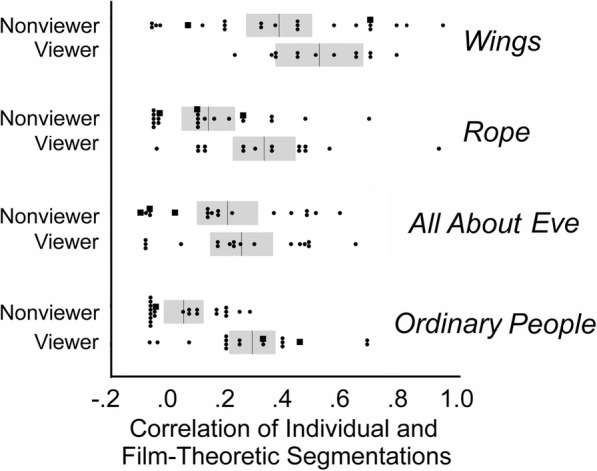
A comparison of the individual subjects’ data (*dots* and *squares*) expressed as correlations of their segmentations against film-theoretic segmentations. Viewers are the students in Experiment 1 who watched the movies and segmented them on written scenarios; non-viewers were the online workers of Experiment 2 who did not see the films but who read and responded only to the scenarios. The 11 non-viewers indicated by *small squares* claimed to have seen their film before. *Faint*, *gray vertical lines* indicate means and *gray bands* indicate standard errors of the means

Each participant was randomly assigned to segment one of the four movies. A total of 201 workers were enlisted, but 77 were then eliminated because they didn’t follow instructions (they segmented the narrative into too many units, they didn’t distribute segmentations throughout the movie, or they didn’t complete the narrative summaries). An additional 38 were eliminated because we believed they rushed through the task (we retained only those who took at least 10 min to complete the segmentations). This left us with 86 usable response sets—23 for *Wings*, 22 for *Rope*, 18 for *All About Eve*, and 23 for *Ordinary People.* The reported average age of these non-viewers was 35 years with a range from 18 to 69, and with 42 reporting to be male, 39 female, and five not reporting. While 11 of our 86 participants reported having seen their movie before (two for *Wings* and three each for the others), we included their data due to the fact that they were unlikely to have seen the movie recently, and were therefore unlikely to have retained a thorough memory of the narrative. Those viewers are noted in Fig. 5.

## Results

The non-viewers offered slightly fewer segmentations (4.28) than did the viewers (4.74, *t* (147) = 3.15, *p* = 0.002), although this difference seems relatively small and with unclear impact. More importantly, shown in Fig. 5 are comparisons of the correlations for non-viewers’ and viewers’ responses with the film-theoretic segmentations. The viewers’ responses matched the film-theoretic segmentations only slightly better than those of non-viewers for *Wings* (*t* (34) = 1.48, *p* = 0.15) and for *All About Eve* (*t* (33) = 0.65, *p* = 0.52), but matched them better for *Rope* (*t* (36) = 2.74, *p* = 0.01) and for *Ordinary People* (*t* (38) = 4.53, *p* < 0.0001). An aggregated mixed-model analysis of correlations with film-theoretic divisions across the four films within viewers and between films for non-viewers revealed a difference between groups (*t* (38.2) = 3.65, *p* = 0.0008, *d* = 1.18). In a post-hoc analysis after inspecting Fig. 5, we also found it impressive that 27 of the 86 non-viewer/film-theoretic segmentation correlations hovered around zero (including 7 of the 11 non-viewers who claimed to have seen their movie before), whereas only 6 of the 57 viewer/film-theoretic correlations did so.

It should also be noted that the non-viewers’ responses, as we suspected, were not random, but correlated with the film-theoretic segmentations for three of the movies. One-sample tests revealed reasonable segmentation ability for *Wings* (*t* (22) = 5.8, *p* < 0.0001), *Rope* (*t* (21) = 3.17, *p* = 0.0046), and *All About Eve* (*t* (17) = 3.84, *p* = 0.0013). Only the non-viewer response patterns for *Ordinary People* (*t* (22) = 1.83, *p* = 0.08) were generally unstructured.

It occurred to us that some of this “above chance” segmentation performance of non-viewers might be due to surface linguistic cues—particularly words that signal time or location changes. However, we found little evidence of this. For example, “meanwhile” never appears in the scenarios, “later” occurs at a major boundary once out of the seven times it appears, “next” occurs at a boundary one out of six times, and “then” once out of 17 times. We also tracked overt changes in locations (going from inside a room to outdoors, a new city mentioned, going from one activity to another when those can only be done in different locations, etc.). We found ten of these corresponded to major boundaries, but there were 79 other such location changes in the scenarios overall. Our best guess, then, is that many of the non-viewers really did understand aspects of the structure of the narrative from reading the scenarios; they just didn’t generally perform as well as the viewers.

## Conclusions and a speculation

We can hardly claim that these seven movies are representative of popular cinema, of English-language cinema, or even of Hollywood cinema. However, they do represent a swath of narrative formats and time periods—a silent movie; a contemporary movie; a network movie; movies with flashbacks; movies with three, four, and five large parts; and movies across a selection of genres—dramas, action films, a suspense film, and a science fiction/puzzle film. And this small sample is helped by the fact that Bordwell (2006) and Thompson (1999) have claimed that there has been a consistency in the overall narrative structure of popular movies regardless of genre or changes across a century of popular filmmaking. Our results endorse that view.

Thus, although one might rightly complain about (1) our bobtailed selection of movies, (2) the fixed order in which they were viewed, (3) the fact that viewers did not segment the movie while watching it, as is typically done in other event segmentation experiments, and (4) the fact that viewers had access to the scenarios while viewing each movie (in the dark), the results are fairly robust and fivefold.

First and most importantly, viewers were reasonably consistent in segmenting movies into large parts, and with no substantial individual differences. This provides evidence for large-scale events in movies.

Second, those segmentations comport well with film-theoretic segmentations by professionals in four cases (*Wings*, *Grand Hotel*, *All About Eve*, and *Source Code*), with those we provided in advance following film-theoretic guidelines for three others (*Passage to Marseille*, *Rope*, and *Ordinary People*), and with no noticeable differences between the two sets. To be sure, the overall results are far from showing uniform congruence among viewers, but across participants these segmentation findings are roughly of the same strength as those for segmenting smaller events (e.g., Zacks, 2004).

Third, the segmentations of viewers in Experiment 1 match the film-theoretic segmentations generally better than non-viewers in Experiment 2 who had access only to the scenarios.

Fourth, as shown in Table 2, the boundaries of these large-scale events are firmly related to the boundaries of scenes, the next smaller scale events. Thus, the relation between the two seems meronomical.

Fifth, there is no strong evidence that viewers used anything but cognitive resources to do their segmentations. To be sure, five movies (*Wings*, *Grand Hotel*, *Passage to Marseille*, *All About Eve*, and *Source Code*) had surface cues to some segmentations that were provided by the filmmakers in terms of intertitles, fades, dissolves, or other salient transitions. But in one case (*Source Code*) viewer segmentations did not reliably correlate with those cues, in another (*Grand Hotel*) the correlation was weak, and in the other two movies (*Rope* and *Ordinary People*) there was no surface information whatsoever that could help.

The idea that cognition alone would be the basis of these segmentations fits with the general schema of event segmentation theory (Zacks & Swallow, 2007). That is, more fine-grained segmentation is typically done bottom-up on the basis of perceptual information (like motion), but more coarse-grained segmentation is done top-down on the basis of conceptual features (like an agent’s goals, and here perhaps the filmmakers’ goals). The event segments researched here are much larger than those typically discussed in the event processing literature. On the basis of these results it makes sense that these large parts in movies can be segmented without needing lower-level perceptual information, and done on the basis of the organization of the many event models (or situation models; Zwaan & Radvansky, 1998) built up over the course of watching a movie.

Finally, and still missing, is any firm understanding for why these larger units should be roughly 20 to 35 min in duration. In this sample they range from 20 to 38 min except for two shorter climaxes, one in *Passage to Marseille* (13 min) and the other in *Source Code* (18 min). Bordwell (2006) and Thompson (1999) both noted that climaxes are often the shortest segments in movies. But, as we quoted earlier (Thompson, 1999, pp. 43–44), is this half-hour span a cognitive constraint?

Perhaps the only cognitive domain relevant here is that of vigilance, the measure of sustained attention over prolonged periods of time (see Parasuraman, 1986), typically in the context of detecting rare targets. Individual differences in this domain are large, and many experiments have measured vigilance during tasks of high stress, such as those for radar operators, air-traffic controllers, and TSA inspectors. Nonetheless, performance falloff (called vigilance decrement) occurs in all situations, including low-load tasks (Frankmann & Adams, 1962), particularly after about 15 to 30 min (Grier et al., 2003; Parasuraman, 1986).

Popular movies, particularly contemporary ones, tend to have relatively complex narratives and narrational structures, not rare targets, but sustained attention is nonetheless a prerequisite. Understanding movies is not always easy, particularly when a brief moment of dialog can quickly change the direction of the plot. Difficulty in sustained attention to a narrative may account for some of the decline in movie watching by older adults (Armstrong & Cutting, 2017).

So here is our conjecture: narrative change, such as that which occurs at a boundary between larger-scale narrative events studied here, may sufficiently freshen the task of understanding so that the viewer can better sustain attention for a new period of 25 min or more. The idea here is that when the narrative goes in a different direction, the viewer has to work harder. Perhaps it is not a coincidence that, in a vigilance task, Thomson, Smilek, and Besner (2015) showed that making a task harder *reduces* the vigilance decrement—that is, people sustain their attention better. Borrowing an idea from Berlyne and Parham (1968) in experimental aesthetics—a domain likely closer to movies than to vigilance—Thomson et al. (2015), p. 387 suggested:

It may therefore be the case that by increasing the perceptual variability of critical targets in a vigilance task, one might arrive at a situation in which the novelty of the task persists for longer, thus holding attention and reducing performance decrements.

Perhaps it is the variability in the movie narrative, particularly at boundaries between large-scale parts, that keeps viewers interested. Indeed, in terms of cross-cutting scenes in narratives, there is evidence that movies have become more complex over the last 70 years (Cutting, 2019b). Moreover, the greater motion, the greater change in luminance, and the greater range of shot durations that typically occur in the climax (Cutting, 2016)—in addition to bringing the story to a close – may help to sustain attention at the end of a nearly two-hour movie experience.

### Summary evaluation

We began this article with an overview of the meronomical organization of many of the arts. We briefly discussed music, literature, theater—for which the evidence seems quite obvious—but also dance, and even aspects of movies. These all have units within units sharing boundaries. Thus, the results here may not seem to be much of a surprise. Essentially, we’ve added psychological evidence to the theoretical notion of a unit intermediate in size between that of scenes and sequences on the one hand and whole movies on the other. This can rightfully be seen as an incremental addition, but not one foundational importance.

We would agree, but we wish to push back somewhat on this simple evaluation. For over a century, filmmakers of popular movies have perfected what is called *continuity editing*. This is a film style that deliberately attempts to elide across, if not completely hide, boundaries among all narrative units. The typical goal of popular filmmakers is to make the progression of events in a movie as seamless as suits the narrative. Thus, cuts between shots are often hard for viewers to detect because of matching on action (Smith & Santacreu, 2016), for example, with leftward motion at the end of one shot followed by leftward motion in the next, within or across scenes; audio coverage (Shimamura, Cohn-Sheehy, Pogue, & Shimamura, 2015) and sound overlaps (called split-edits; which include J-cuts, where the audio track of the first shot of the next scene begins before the last shot of the previous scene is finished, and L-cuts, where the audio lags from the last shot in the previous scene into the first shot of the next scene); and various kinds of *hooks* (Bordwell, 2008), including sound bridges (where the sound of the last shot in one scene is mimicked by the sound of the first shot in the next), graphic matches (where the aspects of the geometric layout of the ending image in one scene is mimicked by that of the first shot of the next), audio to visual juxtapositions (mention in a dialog about a particular book, immediately followed by an image of that book), and vice versa. All of these devices are used to knit scenes together, hiding boundaries by perceptual and cognitive tricks. Thus, psychological evidence that movie viewers can retain larger-scale narrative information in the face of continuity editing is welcome and not a foregone conclusion.

In addition, there are several film-theoretic concerns. Not all film theorists believe that there really should be any psychological reality to concept of an act in a movie. For example, one film glossary suggests: “Since screenplays never show act breaks, an ‘act’ is really a theoretical concept” (August, n.d.). Our data contravene this idea. In addition, our data inform the discussions about two film-theoretic controversies: three (Field, 2005) versus four (Thompson, 1999) large-scale narrative units, and the variable number of such units depending on the length of the movie (Thompson, 1999). Pretty clearly our data support the notion of four such units for average-length movies, three for shorter ones, and five for longer ones. But finally, our viewers and non-viewers alike seemed quite attuned to an early turning point within the first large-scale unit, the setup. We have no evidence that they treated this boundary any differently than the others.

## Data Availability

All data and scenarios are available by contacting JEC and will be posted on a website.

## Appendix

### Movie synopses

Our synopses of the movies follow; paragraphing corresponds to the film-theoretic, larger narrative segments—setup, complication, development, and climax. *Rope* and *Source Code* have no development section, and *All About Eve* has two development sections. Epilogs, if any, are included within the climax paragraphs after long dashes. Numbers in parentheses indicate the location of segmentations of at least two viewers as a proportion of the total number of viewers of that movie. The sentences in these synopses do not correspond to the numbered entries in the scenarios, which can be found at http://people.psych.cornell.edu/~jec7/data.htm.

#### *Wings* (1927)

Two young men from the same town—Jack an auto mechanic and David from a wealthy family—are rivals for Sylvia. She likes David but tolerates Jack’s attention. Mary, Jack’s neighbor, is attracted to him but he only thinks of her as a friend. Jack and David enlist when World War I breaks out. They report to training (0.13), initially as rivals but become fast friends and eventually pilots. Mary also enlists (0.25).

In France, Jack and David go on their first air patrol and engage German planes. Both down enemies, but Jack is forced into a controlled crash (0.35), fortunately near Allied lines, and returns to the air station. Later, unbeknownst to Jack and David, Mary drives a medical supply truck and is caught in a French town under a German bombing mission. Jack and David take off in counterattack. Separately, they vanquish the enemy, and Mary cheers Jack’s heroics. Both pilots are given awards of valor (0.45; intermission).

Later, celebrating in Paris, Jack and David become drunk at a cabaret, but the war is escalating, and all pilots are suddenly called to return. Mary is in Paris too, hunts down Jack, dresses as a chorus girl, pries him away from a French woman, and takes him upstairs to sober him up (0.57). Out of uniform she is compromised and sent back to the US by military police. Jack is returned to duty (0.66).

Later, Jack and David study maps, talk about Mary, and have an unresolved dispute about Sylvia just as they need to scramble to get airborne. Jack shoots down a dirigible as David protects him from enemy planes. Jack returns but David must make a forced landing behind enemy lines. A message is delivered that David is dead (0.74) and Jack takes off on a solo mission of revenge. Meanwhile, David escapes, steals a German plane and flies back. After doing damage to the enemy, Jack sees the German plane and engages it, shooting it down (0.87), and David crashes into a French farmhouse. Jack lands nearby and discovers his mistake just before David dies (0.92). -- -- Later, Jack collects David’s gear and returns home to a parade in his honor (0.95), weeps in apology to David’s parents, and reunites with Mary whom he realizes he should have favored all along.

#### *Grand Hotel* (1932)

Serially, five hotel guests make phone calls about their distress. Later, upstairs and overlooking an atrium (0.03), Flaemmchen (Flaem, a part-time stenographer) and Preysing (Prey, an industrial magnate) arrange for a later meeting for dictation (0.09). The Baron (desperate for money) meets Kringelein (Kring, a terminally ill accountant who wants to splurge at the end of this life). Flaem, Kring, and the Baron talk (0.14). Flaem and the Baron flirt and agree to meet at the bar the next evening. Meanwhile, Grusinskaya (Gru, a performance-weary Russian ballerina) has checked into the hotel with her entourage, and claims she won’t dance, but is nonetheless cajoled into performing (0.19). The Baron overhears talk of her necklace, which he then plans to steal, and meets outside with his shady creditor (0.21).

Meanwhile, in his room Prey dictates a business letter to Flaem (0.24) and flirts with her. Outside on the balcony (0.28), the Baron moves past Prey and Flaem to Gru’s room to steal her necklace and enters by the window. He takes the necklace, but Gru returns (0.30) and he hides in a closet. He becomes entranced, he emerges, they talk all night and fall in love; he returns the necklace. Elsewhere, Kring and the hotel doctor return from drinking (0.37). Later the next day, Prey (0.40) tries to negotiate a business deal with lawyers, and Flaem leaves for the bar. Meanwhile, with the Baron (0.43), Gru becomes radiant and again wants to dance (0.51).

At the bar Kring and Flaem talk. At a meeting Prey lies to the lawyers about his financial situation and must go to England to cover his now-assured losses. The Baron enters the bar. Flaem and the Baron dance and she notices, and he admits to, his change in affections. Prey enters the bar (0.59), and Prey and Kring (whom we learn is Prey’s accountant, a fact then also revealed to Prey) argue. Later, outside the bar, Prey proposes a well-paid tryst in England to Flaem (0.65), mixed with his business, which she accepts. The Baron holds off his creditor and then sends Gru off to dance, saying he will meet her later at the train station so they can depart together to Italy. In need of money, he proposes a card game to a number of people. Kring, a novice at cards, wins big; the Baron loses. Kring becomes drunk, faints, and the Baron helps take him to his room (0.74). There the Baron steals, but then returns, Kring’s wallet when Kring realizes it is gone (0.76).

Desperate for money, the Baron leaves, meets Flaem on her way to Frey’s room. Meanwhile, Gru returns from a brilliant performance and looks for the Baron. Prey and Flaem are in Prey’s suite, and the Baron enters Prey’s bedroom from the balcony to steal his wallet. Prey catches then strangles him (0.83). Flaem screams, escapes to Kring’s room, and Kring goes to Prey and accuses him of murder. The commotion raises the attention of the hotel guests and staff (0.88). The police arrive, Prey is handcuffed, and they depart. Meanwhile, Kring returns to his room and to Flaem. With his new earnings, they talk about a trip to Paris together and book it. Gru and her entourage leave for the train station. Kring and Flaem also leave. New visitors arrive at the hotel, and the hotel doctor says “Grand Hotel. Always the same. People come. People go. Nothing ever happens.”

#### *Passage to Marseille* (1944)

Over Germany, planes of the Free French Air Force bomb a chemical plant. Returning to England over France a bombardier (Matrac) drops a note to his wife and son (0.03). The next day, a reporter is taken to a secret airbase in the south of England to learn about the French air effort. He meets Freycinet, its commander, they talk, and then tour the installation. On the tarmac the reporter is impressed with the bombardier (Matrac), who goes out on another mission. After returning to the command post, Freycinet tells the story of Matrac (and the first-level flashback begins, 0.13). On board the *Ville de Nancy* and over dinner, Freycinet, Duval (a French officer allied with Vichy), the Captain, and others discuss the progress of the war. They pass through the Panama Canal. Later, they discover five men (including Matrac) in bad shape, afloat in a makeshift boat off Guiana. Duval is suspicious. When the five recover they are interviewed by Duval (0.29), with the Captain and Freycinet present. They later tell Freycinet of their troubles (0.33, and the second nested flashback begins).

Four of them were, for different reasons, in a French prison camp in Guiana and escaped with the help of an old butterfly collector, Gran’père. There, they tell Gran’père of a fifth patriot, Matrac (and the third nested flashback begins). Matrac and his soon-to-be wife run a resistance newspaper in France, whose office is raided. They escape and hide from authorities, but are eventually caught and Matrac is sentenced (the third flashback ends). Back in Guiana (0.56), all wait for his release from solitary and then the five escape by night. The boat is too small for six and Gran’père decides to stay behind (0.70, the second flashback ends).

Back on ship (0.70), the radio operator receives a message of the Munich Armistice; the French have capitulated. The Captain plans to divert to England, and asks Freycinet if Matrac can be trusted (0.73). But Duval and his men take over the ship. A fight ensues and the Captain, Matrac, and the others seize back control, but the radio operator has sent for Vichy/German help. A plane bombs and strafes and the ship folk fire back. Matrac eventually downs the plane near the ship and shoots the survivors, much to the captain’s consternation. Victorious but damaged, the ship sails back to Europe (0.89, the first flashback ends).

Back in England, the reporter and Freycinet wait for the return of the planes on bombing runs over Germany. All land but the one with Matrac. Disabled and with Matrac and others injured, the plane cannot drop a birthday message to Matrac’s son. The plane lands and Matrac has died (0.96). -- -- A cliffside burial and tribute is led by Freycinet (0.98), reading Matrac’s letter.

#### *Rope* (1948)

Brandon watches his friend, Philip, strangle David Kentley. Together, they put his corpse in a living-room chest. Brandon goes to his kitchen and pours champagne to celebrate. Mrs. Wilson, Brandon’s housekeeper, arrives (0.17) and prepares for the upcoming cocktail party, where food will be served on the chest. Kenneth, a high school chum, arrives (0.22), as does Janet (0.25), Kenneth’s former girlfriend but now engaged to David. Mr. Kentley (David’s father) arrives with his sister-in-law, Mrs. Atwater (0.29). Philip plays piano and, finally, Rupert (former teacher of Brandon, Philip, David, and Kenneth) arrives (0.35).

Brandon prattles on, telling a story about a chest. Rupert notes that Brandon always stammers when excited, and that a chest always turned up in stories that he liked. All serve themselves food and Brandon recounts a story of Philip strangling chickens (0.42). A conversation follows with Brandon and Rupert approving of selective murder, with Mr. Kentley upset at the Nietzschean implications. Mr. Kentley, who is interested in Brandon’s first editions, goes off with Brandon and the others while Kenneth and Janet talk awkwardly. Later, all notice that David hasn’t yet come. Rupert and Mrs. Wilson talk about the oddness of the party (0.56). Rupert then talks to Philip, who is playing the piano practicing for a concert; and Philip later tells Brandon that Rupert is on to something. Later, the guests begin to leave and Brandon gives Mr. Kentley the first editions bound with the rope that strangled David (0.63). Mrs. Wilson clears the chest and almost opens it (0.71).

After all are gone Brandon congratulates himself and Philip. Mrs. Wilson leaves; the phone rings and it’s Rupert, who “forgot” this cigarette case (0.75). Brandon gets a gun. Rupert returns, has a drink, and imagines how the two might have killed David. He then reveals the rope he obtained from Mr. Kentley. After a struggle and with the gun Philip shoots and grazes Rupert’s hand. Rupert looks into the chest and is appalled (0.93), but also by the fact that, after an explanation by Brandon, the two murderers had taken his high-school seminar words about superior beings too literally. Rupert shoots the gun out the window, and they all wait in silence for the police.

#### *All About Eve* (1950)

At an awards dinner, Eve Harrington is about to receive an award for best Broadway actress. In a voiceover Addison DeWitt, a theater critic, introduces the main characters, lauding praise on Margo Channing, considering her a true actress. Karen Richards (Margo’s best friend) starts a new voiceover about how they all met Eve (0.04, and the flashback begins). Eve has watched Margo’s every performance, hangs around backstage, and Karen invites her in to the dressing room. Karen announces Eve, and Eve tells Margo, Karen, Lloyd Richards (the playwright and Karen’s husband), the (false) story of her past. Bill Sampson (the director and Margo’s partner) arrives late and needs a ride to the airport to catch a plane to California. Bill and Eve talk about “the theater” while Margo cleans up. Margo and Eve go to the airport and see Bill off (0.20); Eve stays at Margo’s apartment and becomes her assistant (0.22).

Late at night and perhaps a week later, Bill calls Margo to her surprise. During the call she realizes that it is Bill’s birthday and talks about arrangements for a party when he returns. The next day, suspicious of her motivations, Margo confronts Eve who admits that she arranged the call (0.27). Weeks later at Bill’s party, Margo bristles for a verbal fight. Guests arrive (0.31) and Margo becomes drunk (0.35). Upstairs, Eve asks Karen to talk to Lloyd and Bill about understudying Margo (0.38). They both go downstairs for a long collective conversation on the steps about “the theater” with Addison, Bill, and Lloyd. Margo quarrels with everyone and retreats upstairs. Guests leave (0.44).

The next day Margo learns from Addison that Eve is to be her understudy, and she is furious. An altercation breaks out with Lloyd (0.48) and he leaves angrily, leaving Margo and Bill to talk. Bill says he is leaving her. Lloyd, at home with Karen, fumes and goes upstairs. Karen, still on Eve’s side, plots. Margo, Karen, and Lloyd go away for the weekend and, because Karen cut the gas line, they become stranded (0.58). Margo misses her performance and Eve performs well (0.62).

Addison goes to meet Eve after the play, but she is with Bill (the director). She propositions him and he rejects her, saying he is still in love with Margo. Addison has overheard the conversation and, after Bill leaves, he enters and talks to Eve (0.64). He asks questions about her past and reveals that her previous story is a sham (although she doesn’t yet realize that he knows this). Addison writes a glowing review of Eve’s performance, berating Margo. Karen and then Bill rush to Margo to console her. Later (0.69), Lloyd talks to Karen about a new play, one for Eve. Karen becomes irate, but Margo calls and they arrange a dinner for four. At dinner (0.72) Bill announces that he and Margo are getting married. Addison and Eve are also in the restaurant, and Eve sends a note to Karen to meet her in the ladies’ room (0.73). Eve threatens to blackmail Karen (about the cut gas line and arranging critics to be at her understudy performance) if Karen doesn’t convince Lloyd to have her for the lead in his next play. Karen returns to Lloyd, Bill, and Margo, and Margo announces that she is through with ingénue roles and doesn’t want to be in Lloyd’s new play, leaving the role open for Eve. Karen laughs. Eve, Bill, and Lloyd then rehearse the new play (0.82).

Later, Addison and Eve are in New Haven for the out-of-town opening. They verbally tussle (0.84), and Addison reveals all the lies she has told and tells Eve that he is now in control of her life. Eve performs well and (end of the flashback, 0.93) the story reverts to the award ceremony. Eve gives a short speech, thanking all of her “friends” and promising to return to the theater after a stint in Hollywood (thus escaping Addison). She is congratulated by many but doesn’t go to the after-party. -- -- Addison drives her to her apartment building, Eve enters her apartment and discovers a young woman, Phoebe, there (0.96). Phoebe (like Eve at the beginning) is awestruck and wants to become a famous actress. Addison returns to deliver Eve’s award which she had left in the car. Phoebe takes the award, puts on Eve’s robe, and bows before her reflection in many mirrors.

#### *Ordinary People* (1980)

Conrad and other students sing at high-school choir practice. Later, at night he awakens in cold sweats. Perhaps at the same time Beth and Calvin, his parents, watch a community theater production, talk some with friends, and drive home. Calvin looks in on Conrad and they discuss the idea of him seeing a therapist. Next morning, Conrad skips breakfast and is picked up for school by three friends. Along the way he has a vision of a graveyard. Conrad is listless in class, alone outside at lunch, and finally calls the therapist (Berger). He is unfocused at swimming practice (0.11), and that night has several more bad dreams. He sees Berger (0.12) and talks about “self-control”. At dinner he tells his parents that he saw the therapist. Calvin is pleased, Beth not so much. The next day after choir Jeanine (who stands in front of Conrad) tells him she admires his tenor voice. Later (0.20), Beth comes home and stares into Buck’s undisturbed room (Buck was Conrad’s older brother, who died). She sits down and looks at his many trophies and pictures. Conrad arrives home, and he and Beth surprise each other but without really making contact. Later, at a cocktail/birthday party, Calvin tells a friend that Conrad is seeing a therapist, which Beth overhears. On their way home Beth is outraged, pleading for family privacy (0.26).

Conrad returns to Berger and talks about his “feelings” and quitting swimming. Later, he meets Karen (who was in hospital with him) at a soda shop and they talk. Karen seems upbeat but she is not seeing a therapist. They promise to stay in touch. Later (0.33), Conrad sits at home in the backyard; Beth joins him. They talk, but disagree over Buck having wanted a dog, and Conrad barks at Beth. Beth goes inside, then Conrad. He almost apologizes but Beth is called away to the phone. Beth laughs and, in a flashback, Conrad remembers how warm she was when Buck was alive. Conrad then sees Berger, and says he didn’t know how to feel when Buck died (0.36). Meanwhile, Calvin returns home on the train having thoughts of joyous tumult when the kids were younger but also of Conrad trying to kill himself. Conrad quits the swim team (0.41), then sees Berger and “feels lousy” (0.43). At home, Conrad’s grandparents visit and they try to take pictures. Beth and Conrad have difficulty standing next to one another, and Conrad lashes out (0.48). After choir Jeanine reinforces her appreciation of Conrad’s voice. They talk and walk to her bus; he sings walking home. In his bedroom he tries to call Karen, who isn’t there, then calls Jeanine asking for a bowling date. Later, Calvin and Conrad bring home a Christmas tree and set it up. Beth enters upset that she had to learn that Conrad quit the swim team from a friend. All explode in anger. Conrad goes upstairs; eventually Calvin follows and they talk (0.60).

Later, Conrad and Berger talk about forgiveness and limitations. Later still, Calvin is running, falls, and thinks. Next, he goes and talks to Berger. Afterwards, Calvin goes home and sits in his car in the garage. Beth comes, they talk about a detail from Buck’s funeral, and hug. Later, they meet at a shopping mall. Beth discusses getting away for Christmas without Conrad; Calvin is not convinced and wants family therapy, but demurs. They decide to go see her brother and sister-in-law in Texas. Later (0.69), Conrad and Jeanine go bowling and she throws gutter balls. They sit in a booth and she asks him about trying to kill himself. He starts to answer but is disrupted by his friends entering the bowling alley. Jeanine laughs nervously, and apologizes on their way home. Meanwhile, Beth and Calvin land in Texas and golf with her relatives. Conrad attends a swim meet, stays after, meeting his former teammates. He is insulted by one and slugs him. The fight is broken up, Conrad retreats to his car, and rebuffs his former best friend. Conrad returns to his grandmother’s house (0.78).

He phones Karen and is told that she committed suicide. Conrad is distraught, goes into the bathroom, looks at the scars on his slit wrists, but finally grabs his coat and runs outside. As he runs he relives fragments of the boating accident, which he survived but Buck didn’t. He goes to Berger (0.81), they talk, and Conrad finally realizes that Buck’s death was not his fault (0.87). Later, Conrad is outside Jeanine’s house early in the morning. She sees him, goes out, they talk and then go in for breakfast in her house. Meanwhile, Beth and Calvin are golfing with her relatives. They explode in anger over Conrad. Returning home on the plane, Calvin remembers good times with Beth and wonders what happened. Home in their living room, Conrad joins them. He hugs his mother but she can’t hug him back. Calvin notices (0.93). That night Calvin is downstairs crying. Beth goes down to see him. He says he doesn’t think he can love her anymore. She goes upstairs, cries, packs, and leaves by taxi. The next morning Conrad finds Calvin in the back yard and they talk.

#### *Source Code* (2011)

Captain Colter Stevens, a helicopter pilot in Afghanistan, awakens on a train to Chicago sitting across from Christina Warren, who recognizes him as someone else, Sean Fentress. He scurries around the train, sees Fentress’s face in the bathroom mirror, is puzzled, and sits back down as the train explodes (0.07). Colter awakens in a dark pod and looks at Goodwin through a small window and is told by her that he is on a mission to find the bomber of the train. Back on the train (0.12), always reliving the same six minutes, Colter (as Fentress) is more comfortable with Christina, and scans the passengers. In the bathroom he finds the bomb, takes off a cell-phone trigger, goes back to the train car, and interviews passengers with phones. The train explodes (0.19), Colter is back in the pod, and talks to Goodwin about what he knows. Goodwin and her boss, Rutledge, send him back (0.21) with the mission to find the terrorist who planted the bomb. Colter and Christina scan passengers together, he kisses her, defuses the bomb, and they exit the train together at a stop to follow a suspect. Colter sees the train explode (indicating a second trigger that he missed). He fights the suspect and falls under an oncoming train, taking him back to the pod (0.33).

The connection with Goodwin is poor and Colter is freezing, but connection is finally made. Rutledge explains that a second, and dirty, bomb set by the same terrorist will explode in Chicago, and that he (Colter as Fentress) needs to find the terrorist. To help him, Goodwin says there is a gun upstairs in the rail car in the conductor’s closet. Back on the train (0.42), he breaks into the closet but is tasered by the conductor and handcuffed to a luggage rack. Explosion (0.46) and he is back in the pod. He is sent back to the train, but not before he sees an Army insignia that may help explain his circumstances. On the train he sketches it from memory, a woman passenger recognizes the insignia (0.48), and Colter infers Rutledge’s location and tries to call him (0.52). Meanwhile Colter (as Fentress) had asked Cristina to find out about Captain Colter Stevens. Christina tells him that Stevens is dead. Colter faints (0.54) and he’s back in the pod. He then goes back and forth—train to pod to train—several times. Eventually, he finds the bomber and follows him to his van and sees the second bomb (0.69). Christina has followed him and the distraction allows the bomber to shoot them both, killing them. Colter is back in the pod, and tells Goodwin about the bomber. TV coverage (0.74) shows the bomber being arrested, but Colter wants to go back one last time to save the people (and Christina) on the train (0.80).

Against Rutledge’s wishes, Goodwin sends him back. Colter defuses the bathroom bomb (both triggers), catches the terrorist, handcuffs him to the same rack, and calls the police about the second bomb. As the 6-min interval elapses for the final time, nothing happens (0.95). Goodwin looks into the pod and sees Colter’s body is missing below the chest. -- -- Meanwhile, Colter and Christina leave the train, go to the Chicago Bubble (where Colter is reflected as Fentress). Back at the lab, the reality of Goodwin and Rutledge is that the bomber was captured without the help of Source Code, but Goodwin receives an email from Colter that this is not the case, and that Source Code works better than anyone thought.

## Footnotes

1 Both are websites evaluating and providing information about movies. Internet Movie Database (IMDb) is at https://www.imdb.com and Rotten Tomatoes is at https://www.rottentomatoes.com. Values can change with time. Those listed in this paper were harvested on 21 December 2018. In addition, the American Film Institute’s rankings are found at https://www.afi.com/100years/movies.aspx.

2 The scenarios for each film can be found at http://people.psych.cornell.edu/~jec7/data.htm.

3 Since different viewers yielded different numbers of segments, we also assessed the results by weighting them by the number of segments reported. Thus, if a viewer generated two segment boundaries, these would be considered twice as important as another viewer’s four segment boundaries. However, this approach yielded nothing different than an unweighted procedure, which we followed.

4 There is an apparent typographical error in Thompson’s (1999) appendix. It lists *Wings*’s development as 37 min in duration, but it can only be 27 min.

5 Pearson product-moment correlations, like other parametric statistics, have problems when a dataset contains many true zeros. The reason is that the distributions become strongly skewed. Such distributions are said to have “zero-inflation” or “zero-clustered data”. In such situations, nonparametric approaches are often suggested, but the number of zeros here typically exceeds even what is appropriate in those cases as well. However, Huson (2007) showed that when 10 to 30% of a sample dataset of 50 entries are zeros (about the set size we have here in most cases), both Pearson and Spearman correlations are underestimated by values of 0.01 to 0.05, with the underestimation increasing with the magnitude of the correlation. It appears that an increase in zeros is like an affine transformation downward of the value of the correlation; but it does not change the ordinality of results with comparable numbers of zeros. As many as 90% of the values in our datasets are zeros and, thus, the underestimation is likely to be even greater. Moreover, we are gratified that having many zeros will underestimate the true size of the effect. Conservatism in statistics is a good thing.

6 We were surprised that there were no segmentations in *Passage to Marseille* when going from the second-level flashback (in Guiana) to the third (in France, 0.44), and only one from France back to Guiana (0.57). However, given that segmentations at every flashback would yield seven sections, placing a transition at every flashback and flash-forward would violate the instructions the viewers were given.

7 Bordwell (personal communication, 7 January 2019). He also suggested that the narrative structure of *All About Eve* is a bit ill-formed and nonconforming to the standard narrative structure because of disputes between the director/writer (Joseph L. Mankiewicz) and the producer (Darryl F. Zanuck), who had the rights of final cut. Mankiewicz wanted the film to be much longer (hence a stronger candidate for five larger parts) but Zanuck cut it down. He discusses some of this in a blog post (Bordwell, 2013).
